# Effects of collagen crosslinking on porcine and human tarsal plate

**DOI:** 10.1186/s12886-019-1254-3

**Published:** 2019-12-16

**Authors:** Sarah W. DeParis, Angela Y. Zhu, Shoumyo Majumdar, Jing Tian, Jennifer Elisseeff, Albert S. Jun, Nicholas R. Mahoney

**Affiliations:** 1 Department of Ophthalmology, Wilmer Eye Institute, Johns Hopkins Hospital, 600 North Wolfe Street, Baltimore, MD 21287 USA; 2 Department of Biostatistics, Johns Hopkins Hospital, Baltimore, MD USA; 3 Department of Biomedical Engineering, Johns Hopkins Hospital, Baltimore, MD USA

**Keywords:** Floppy eyelid syndrome, Crosslinking, Riboflavin

## Abstract

**Background:**

Floppy eyelid syndrome is a disorder in which the tarsal plate is easily distensible and is currently treated with conservative or surgical measures. Human tarsal plate contains type I collagen, which is crosslinked in corneal tissue as a treatment for keratoconus. We hypothesized that collagen crosslinking would similarly stiffen tarsal plate tissue and investigated this in porcine and human tarsal plate specimens.

**Methods:**

Riboflavin-sensitized porcine and human tarsus samples were irradiated with ultraviolet-A light. Porcine experiments were analyzed with gross photographs, anterior segment optical computed tomography (AS-OCT) imaging, and tensile testing. A prospective study of human tarsus was performed on samples from patients undergoing wedge resection for floppy eyelid syndrome and was analyzed with AS-OCT and tensile testing.

**Results:**

73 porcine adnexa and 9 patients (16 eyelids) who underwent wedge excision were included in the study. Grossly, greater stiffness was observed in crosslinked porcine tissue. AS-OCT imaging in porcine tissue showed a distinct hyperreflective band in crosslinked specimens whose area and intensity increased with longer treatment time (*P* = 0.003); this band was also visible in crosslinked human specimens. Tensile testing was performed, but results were not statistically significant.

**Conclusions:**

AS-OCT imaging, which has not been previously described for tarsal plate, showed a characteristic change in crosslinked porcine and human specimens. Tissue stiffness was increased grossly, but changes in tensile properties were not statistically significant. Further study is warranted to determine relevance as a potential treatment for floppy eyelid syndrome.

## Background

Floppy eyelid syndrome (FES) is a disorder in which the upper eyelids are lax and easily distensible and was first described by Culbertson and Ostler in 1981 [1]. Clinically, it is characterized by upper eyelids that easily evert with traction, chronic papillary conjunctivitis, eyelash ptosis, blepharoptosis, and resultant keratopathy [2]. Chronic conjunctivitis is exacerbated by mechanical trauma to the eyelids during sleep, often to a greater degree on a patient’s preferred sleeping side [2, 3]. FES most commonly affects middle-aged men and is strongly associated with obesity and obstructive sleep apnea (OSA) [4]. Additional associations have been reported with keratoconus, dermatochalasis, skin and joint hyperextensibility, hypertension, and developmental delay [2].

Many pathologic changes in FES eyelids have been described, and although nocturnal mechanical trauma and eye rubbing are thought to play a role, it remains unclear if these are the primary etiological factors in the development of FES [1, 4]. It has been hypothesized that a genetic abnormality in collagen, elastin, or both may predispose patients to the development of FES [5]. In addition, pressure induced eyelid ischemia and systemic reperfusion oxidation injury during sleep may contribute to the degenerative changes seen [6, 7].

Normal human tarsal plate provides the structural integrity of the upper eyelid and is not typical fibrous connective tissue or cartilage, but displays some features of both. It consists of fibroblasts surrounded by extracellular matrix, which contains primarily collagens type I and III, elastic fibers composed of elastin and microfibrils, proteoglycans, and glycoproteins concentrated around glandular acini [8, 9]. Several studies have demonstrated normal collagen structure and periodicity in FES [9–11]. However, there is evidence of type I and III collagen accumulation, which may be an adaptive response to repetitive loading of the tissue [9]. In addition, histopathologic studies of FES eyelids have revealed decreased quantity of elastic fibers, change in elastic fiber phenotype from mature elastic fibers to elaunin and oxytalan fibers, and increased levels of matrix metalloproteinases (MMP) that degrade elastic fibers [2, 9, 12, 13]. These changes may also be induced responses due to repetitive stress to the tissue [9]. Tarsal plate fibroblasts have a higher mechanostat set point and decreased sensitivity to mechanical stress, which is proposed to be an adaptive response to greater tissue stress levels in FES [14].

Current treatments for FES include conservative measures such as artificial tears, taping the eyelids closed or wearing a protective shield during sleep [13]. Some patients have improvement of ocular surface symptoms from FES after initiating continuous positive airway pressure (CPAP) therapy for OSA [15]. Surgical treatments include horizontal eyelid shortening and stabilization by wedge resection, plication, or lateral tarsal strip [2, 13, 16]. Despite these interventions, patients may have inadequate symptomatic relief and may require repeated surgical interventions [16]. An effective, minimally invasive method of increasing tarsal plate stiffness could prove useful either alone or in combination with current treatment methods.

Keratoconus is a corneal ectatic disorder in which progressive steepening of the cornea occurs, the normal parallel organization of collagen fibrils is disrupted, and elastic fibers are abnormally distributed [17]. Since it was first described in 2003, corneal collagen crosslinking has demonstrated ability to halt the progression of ectasia with promising long-term disease stability. It has been approved by the U.S. Food and Drug Administration for the treatment of keratoconus since 2016 [18]. Riboflavin, a photosensitizer applied directly to the corneal stromal collagen, is exposed to UVA (ultraviolet A) radiation, inducing bond formation between collagen fibers (primarily type I and III) and increasing corneal stiffness [19, 20]. In addition, a recent study demonstrated significant stiffening of sheep tarsal plate tissue following collagen crosslinking [21].

In this study, we investigated the effects of collagen crosslinking on porcine and human tarsal plate samples. Given the presence of type I and III collagen in both tarsal plate and corneal tissue, we hypothesized that collagen crosslinking may increase the stiffness of tarsal plate. If effective, tarsal collagen crosslinking could be further investigated as a minimally invasive treatment for FES, as no treatments currently exist to prevent pathophysiological progression of this disease.

## Methods

Porcine studies were conducted using fresh porcine cadaveric adenexal tissue retrieved within 24 h postmortem from a slaughterhouse. The slaughterhouse was aware of the intended use of the porcine orbit specimens for medical research but was not asked to give specific consent for the study parameters nor was this required by the IRB who reviewed the study protocol. For human specimens, a prospective study was conducted of patients undergoing wedge excision for floppy eyelid syndrome between October 1, 2016 and April 1, 2018 in a single surgeon’s practice at the Wilmer Eye Institute. After giving written informed consent, patients undergoing unilateral or bilateral wedge excisions for floppy eyelid syndrome were included. The removal of eyelid tissue was in keeping with standards of clinical care, and the tissue would have otherwise been discarded. Patients with a history of prior upper eyelid surgery or with other indications for wedge excision such as neoplasm or trichiasis were excluded. The Johns Hopkins Institutional Review Board prospectively granted approval for this study (No 00116226), which followed the tenets of the Declaration of Helsinki. Informed consent was obtained from each patient for inclusion in the study, which was HIPAA-compliant.

### Porcine collagen crosslinking

A total of 73 fresh porcine adnexa were used in this study. For each experiment, porcine upper eyelid tarsus was dissected free of surrounding tissues. The conjunctiva of porcine eyelids is thicker and less adherent to the tarsal plate than in human eyelids, and was dissected off the tarsal plate completely using blunt and sharp dissection with Stevens scissors. Each porcine adnexa contributed 1 upper eyelid tarsal plate to either the treatment or control group. As porcine adnexa were obtained from the supplier as a unit already removed from the animal, it was not possible to select a contralateral eyelid from the same animal as an internal control. A 30-min riboflavin pre-treatment was then completed for both treatment and control groups, with 1 drop of riboflavin solution applied every 5 min to the anterior surface of the dissected specimens. For the treatment samples, this was followed by collagen crosslinking under UVA light application (370 nm) at irradiance of 8–9 mW/cm^2^ as previously described in prior experiments for corneal tissue for either 30 min or 60 min [22]. The control samples were covered with aluminum foil to block ambient UV light during this time period. During the entire irradiance period, 1 drop of riboflavin solution was applied every 5 min to both specimen groups. Specimens were then stored at 4 °C in 200 uL of balanced salt solution, and either tensile testing or imaging was then performed as below.

**Fig. 1:**
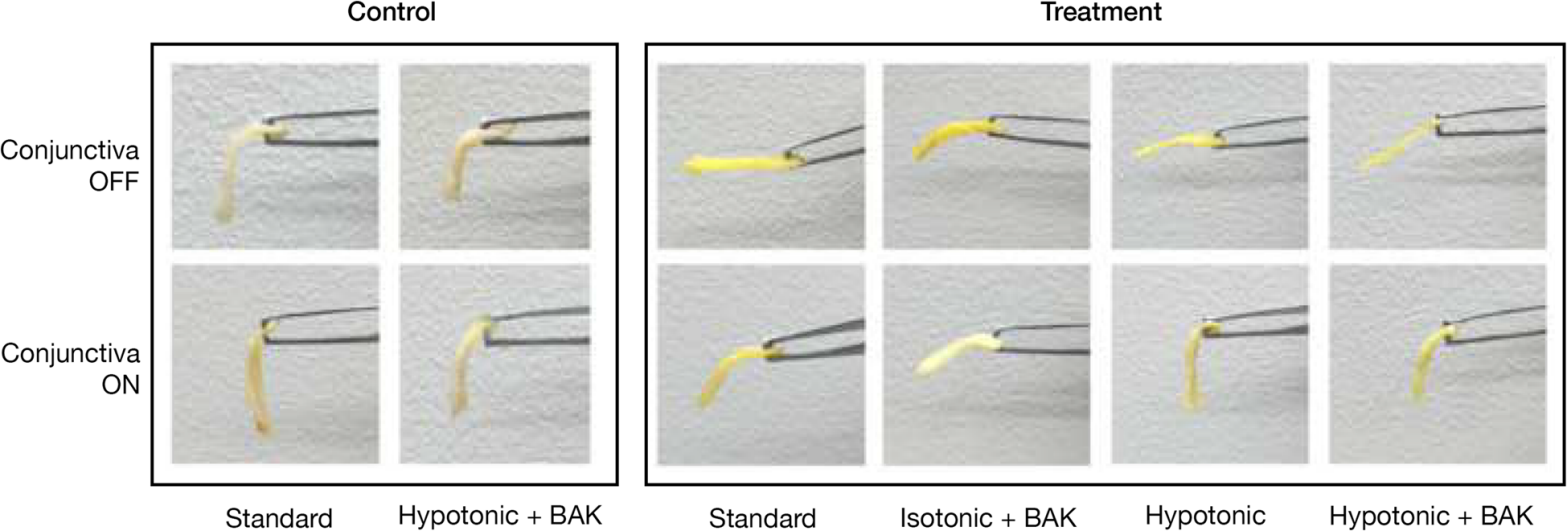
Preliminary Experiment. Gross photographs of porcine tarsal plate after 30-min treatment with standard 0.1% riboflavin in isotonic 20% dextran (“standard”), 0.1% riboflavin in isotonic 20% dextran with 0.05% benzalkonium chloride (BAK) (“isotonic + BAK”), 0.1% riboflavin in hypotonic 20% dextran (“hypotonic”), and 0.1% riboflavin in hypotonic 20% dextran with 0.05% BAK (“hypotonic + BAK”). Conjunctiva was dissected off in one group (“conjunctiva OFF”), and left intact for an additional group of specimens (“conjunctiva ON”). Greater tissue stiffness was observed grossly in treatment specimens as compared to controls, and this effect seemed to be greater in “conjunctiva OFF” treatment specimens, but photographs lacked standardization

In the conventional Dresden protocol for corneal crosslinking, the corneal epithelium is removed and 0.1% riboflavin in isotonic 20% dextran-T-500 is applied as the photosensitizing solution [18]. A preliminary porcine experiment was performed to determine if variations of the corneal crosslinking protocols would have differing effects on tarsal plate tissue. The Dresden protocol riboflavin solution as well as several variations were tested, including 0.1% riboflavin in hypotonic 20% dextran (Sigma-Aldrich, St. Louis, MO, USA), 0.1% riboflavin in hypotonic 20% dextran with 0.05% benzalkonium chloride (BAK) (Sigma-Aldrich, St. Louis, MO, USA), and 0.1% riboflavin in isotonic 20% dextran with 0.05% BAK (Fig. 1). BAK has been shown to increase riboflavin penetration and absorption through corneal tissue, and hypotonic riboflavin solution has been described for crosslinking in thin corneas [23, 24]. In addition, corneal crosslinking has been described with both epithelium intact or with epithelial debridement (“epithelium-off”), with a greater effect seen for epithelium-off crosslinking [25]. In the preliminary porcine experiment, conjunctiva was left in place for one group (“conjunctiva-on”) and was dissected off for a second group (“conjunctiva-off”) (Fig. 1). The 0.1% riboflavin in isotonic 20% dextran solution and conjunctiva-off procedure was selected for all further porcine experiments and for all human experiments.

### Human collagen crosslinking

A total of 9 patients (16 eyelids) who underwent wedge excision were included in the study. Collagen crosslinking was performed on the same day as the patient’s surgery within 8 h of the time of specimen collection, with specimens stored in balanced salt solution at 4 °C until crosslinking was performed. The tarsal plate was dissected free from the surrounding tissues, and the skin and orbicularis were completely removed from the anterior surface. The tarsal conjunctiva of human eyelids is thinner and more adherent, so the posterior surface was debrided with a #15 blade to remove the epithelium. When bilateral specimens were collected, the contralateral side was used as the experimental control. Treatment was applied to the anterior surface of the tarsal plate specimens. The riboflavin solution used was 0.1% riboflavin in isotonic 20% dextran. Pre-treatment with riboflavin was performed for both control and treatment specimens with 1 drop riboflavin administered every 5 min for 30 min. The treatment specimens were then placed under the same UVA light as for porcine specimens for 30 min, and the control specimens were covered with aluminum foil. During the 30-min irradiation period, 1 drop of riboflavin was applied to both groups every 5 min. Specimens were stored at 4 °C in 200 uL of balanced salt solution, and either tensile testing or imaging was then performed within 48 h as below.

### Imaging and “flop” analysis

**Fig. 2:**
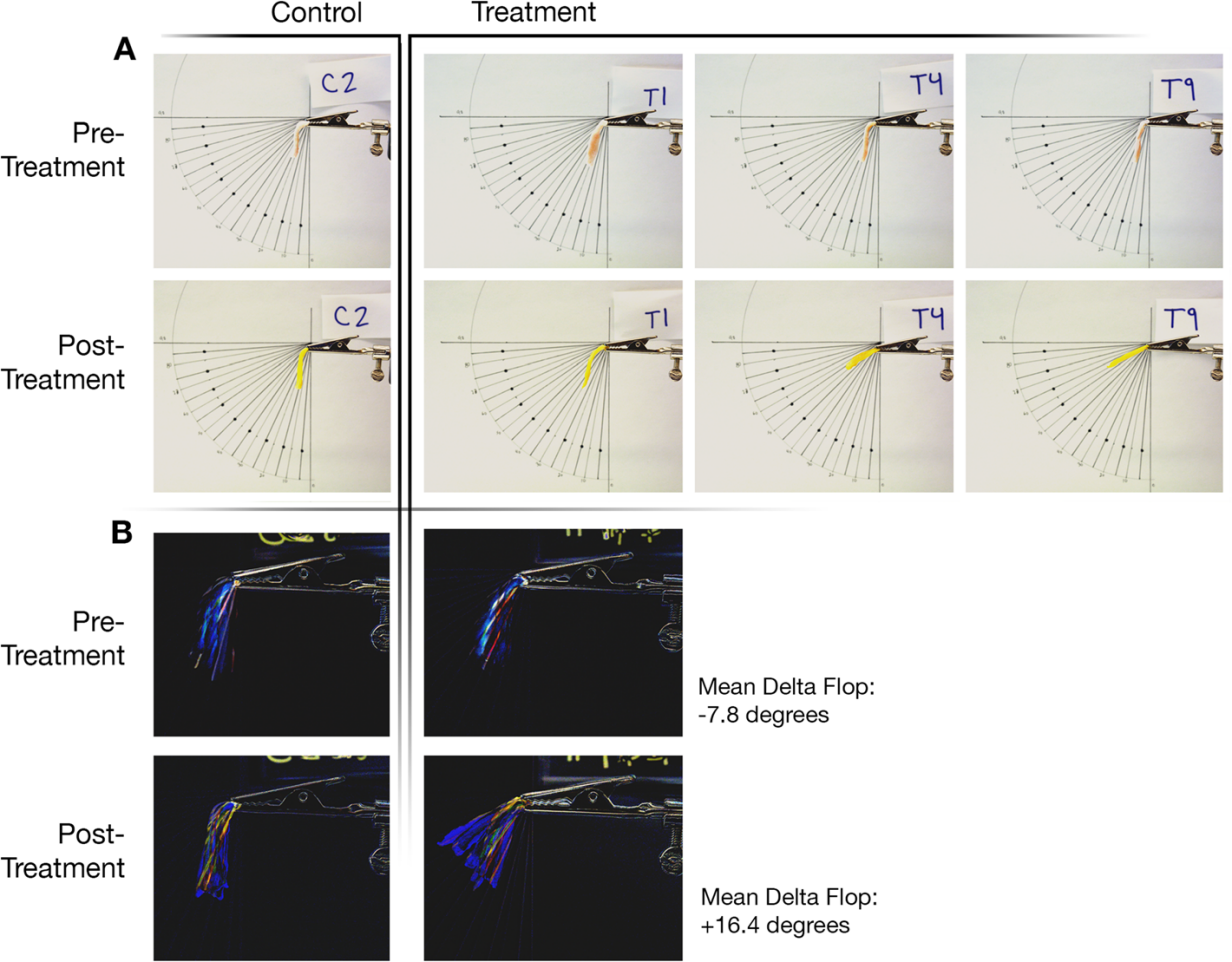
Gross Photographs and “Flop”. (**a**) Gross photographs of porcine tarsal plate specimens using a standardized metal clamp mounted in a fixed location to the camera and a registration image. Images were auto-aligned via position in Adobe Photoshop using the registration marks and then exposure corrected for contrast. Pre-crosslinking treatment and post-crosslinking treatment photographs are shown for representative control (C2), treatment specimen with least “delta flop” (T1), and two treatment specimens with greatest “delta flop” (T4 and T9). (**b**) Composite image of all photographed porcine tarsal plate specimens (10 control, 10 60-min treatment) using a standardized metal clamp mounted in a fixed location to the camera and a registration image, demonstrating a change in “flop” for the post-treatment group. All tested control and treatment specimen images were auto-aligned via position in Adobe Photoshop using the registration marks and then exposure corrected for contrast. The overlay image was created using the Layer Difference Blending Mode. *P < 0.0001

Porcine tarsal plate specimens were dissected in the manner described above and cut to a standard size of 7 mm wide by 30 mm long. All specimens were stored at 4 °C until the time of dissection, and were kept at room temperature from the time of dissection to photographing which was approximately 1 h. Gross photographs of porcine tissue were taken using a tripod-mounted Nikon D7100 DSLR and 105 mm lens (Nikon, Melville, NY, USA). Standardization of gross photographs for “flop” measurement was established using a horizontal metal clasp mounted on a wood frame with the axis of the clamp aligned to 180 degrees (Fig. 2a). The clasp end was positioned 2 mm from the end of the specimen. The 60 min treatment protocol was selected as this demonstrated the most significant changes (wider hyperreflective band) on OCT analysis. Photographs were taken before and immediately after treatment for 10 control specimens and 10 extended (60-min) treatment specimens. Angle of flop as degrees below 180 before and after treatment was measured accordingly from the photographs. The change in angle from before treatment to after treatment, or “delta flop,” was compared between control and 60-min treatment groups (Fig. 2b).

**Fig. 3:**
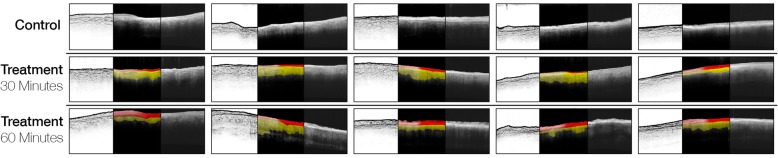
Porcine AS-OCT. Anterior segment optical computed tomography (AS-OCT) of control, 30-min treatment, and 60-min treatment porcine specimens. The entire OCT for each specimen is shown, with unadjusted raw image on the right; black and midtone standardization, image inversion, and Adobe Photoshop Find Edges tool applied on the left; and reinverted image with the two measured areas shown in center, with the hyperreflective band highlighted in red and the deeper tissue highlighted in yellow. The area of the hyperreflective band (center panel, red) was significantly wider in 60-min treatment specimens compared to 30-min treatment specimens (*P* = 0.003)

**Fig. 4:**
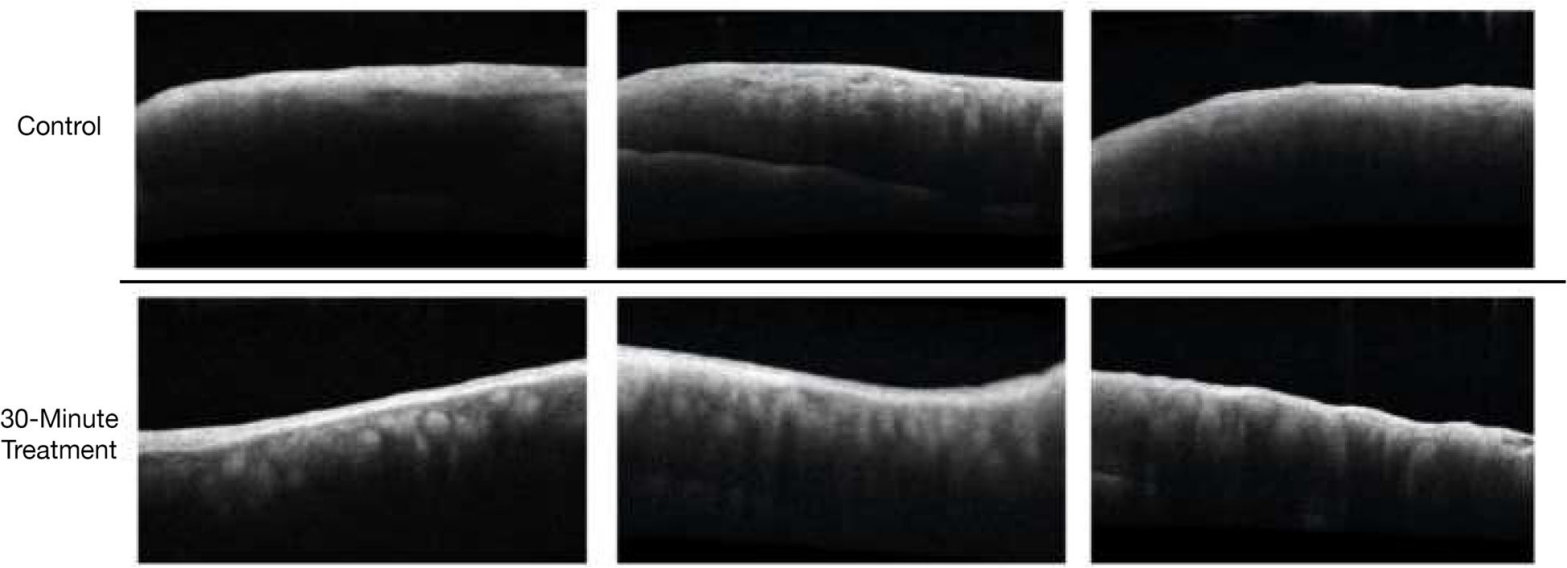
Human AS-OCT. AS-OCT of bilateral upper eyelid human tarsal plate specimens from 3 patients with floppy eyelid syndrome demonstrating a hyperreflective band in the treatment specimens after a 30-min collagen crosslinking protocol. The hyperreflective band is not observed in the un-treated contralateral eyelid tarsal plate specimens, which served as controls

Anterior segment optical computed tomography (AS-OCT) of porcine and human tarsal plate specimens was performed within 48 h of crosslinking with the Zeiss Cirrus spectral domain HD-OCT device (Carl Zeiss Meditec, Dublin, CA, USA) using an anterior chamber lens. For porcine specimens, Adobe Photoshop (Adobe Systems, San Jose, CA, USA) was used to measure the area of the observed hyperreflective band with the following protocol (Fig. 3): The image coloring and tonality was standardized and compressed in two steps identically for all images: 1) the negative space of the scan was set to the black point of the image using the Levels tool and 2) the midtone point was set to 0.25. The image was then inverted and the Find Edges filter was applied to highlight the boundary of the hyperreflective band. In the central 150 pixel wide area of each scan, the Cluster Pixel Selection tool (tolerance 20) was used to highlight the areas of the hyperreflective band (Fig. 3, red) and the homogenous layer of the scan (Fig. 3, yellow). The area edges were smoothed with the Selection Smooth function with a factor of 2 and then the Measurement panel was used to calculate the area, average height, greyscale brightness, mean, minimum and maximum for each band. Comparison was then made between the 30- and 60-min treatment groups for the height and area of the hyperreflective band and the greyscale differences within specimens for the two measured areas (red and yellow). For human specimen AS-OCT, no measurements or adjustments were made (Fig. 4).

### Tensile testing

Porcine and human specimens underwent collagen crosslinking with the 30-min treatment protocol as above. Uniaxial tensile testing was then performed using a MTS Criterion 43 Tensile Tester (MTS, Eden Prairie, MN, USA). Pig and human tarsal samples were each cut into uniform 5 mm strips. Tissue thickness and central width for each strip was measured using a Vernier caliper (Mitutoyo, Japan) and entered into the software. The ends of individual tarsal sample strips were then placed between sandpaper and affixed using super glue to minimize slipping during the tensile test. Strips were placed in the metal clamps of the tensile tester with a sample test length of 5 mm. Pre-loading was done at 5% as is standard for soft tissue. Samples were uniaxially stretched at 0.2 mm/s until failure using a 100 N load cell. Load/displacement measurements were recorded and analyzed. Tensile strength as indicated by peak stress and Young’s elastic modulus were measured.

### Statistical analyses

Statistical analyses were performed using SAS software. Statistical differences of means were calculated using student’s t-test with *P-*values less than 0.05 considered significant.

## Results

In the preliminary porcine experiment, several different variations of riboflavin solution were tested as well as conjunctiva-off and conjunctiva-on procedures. Gross photographs taken before and after crosslinking showed a clear change in tissue stiffness, with greater stiffness of treatment specimens as compared to control specimens (Fig. 1). There was apparently greater effect in the conjunctiva-off group.

For further porcine studies and all human studies, the Dresden protocol riboflavin solution of 0.1% riboflavin in isotonic 20% dextran solution was selected, and the conjunctiva-off procedure was used. In standardized gross photographs of control specimens and 60-min treatment specimens mounted on a horizontal clamp (Fig. 2a), the change in specimen angle before and after crosslinking treatment (“delta flop”) was significantly different between control (mean − 7.8 degrees) and treatment (mean 16.4 degrees) specimens (*P* < 0.0001, Fig. 2b).

AS-OCT imaging of 15 porcine specimens demonstrated the appearance of a characteristic hyperreflective band at the anterior surface of 30-min and 60-min treatment specimens that was not present in controls (Fig. 3). Intraspecimen assessment of greyscale intensity within the hyperreflective band relative to the deeper tissue confirmed this band was brighter in maximum intensity (*P* = 0.014) and average intensity (*P* = 0.039) in the 60-min compared to the 30-min treatment groups. The area of the band was significantly greater in the 60-min compared to the 30-min treatment group (*P* = 0.003, Fig. 3). There was a trend towards greater height of the band in the 60-min treatment group, this was not statistically significant (*P* = 0.056). A similar hyperreflective band was also observed in human specimens (Fig. 4).

For tensile testing in porcine tissue, 13 control and 13 treatment specimens were tested. In human tissue, 4 control specimens and 6 treatment specimens were tested (Table 1). There was no significant difference in the Young’s modulus for either porcine or human tissue (*P* = 0.90 and 0.36, respectively).

**Table 1:** Tensile Testing

Stress-strain measures	Control samples		Treatment samples		
	*Mean*	*SD*	*Mean*	*SD*	Two-tailed *P*-value
Porcine tarsal experiments					
Peak Load (N)	28.53	12.01	32.75	14.25	0.42
Peak Stress (MPa)	4.19	2.00	5.31	3.04	0.28
Strain at break point (in/in)	1.70	1.52	3.02	2.54	0.16
Young’s modulus (MPa)	8.60	7.51	8.99	7.72	0.90
Number of Samples	*13*		*13*		
Human tarsal experiments					
Peak Load (N)	10.43	1.42	11.13	1.96	0.54
Peak Stress (MPa)	2.15	0.47	2.82	1.27	0.28
Strain at break point (in/in)	0.48	0.07	0.49	0.16	0.82
Young’s modulus (MPa)	6.32	2.23	7.82	2.61	0.36
Number of Samples	*4*		*6*		

Tensile testing in porcine and human specimens shows no significant difference in the Young’s modulus or peak stress for either group (*P* > 0.05)

## Discussion

Given the current lack of minimally invasive, curative interventions for FES, our goal was to investigate collagen crosslinking as a putative method for increasing tarsal plate stiffness in both porcine and human specimens.

In our study, the crosslinking protocols used are comparable to accepted protocols for corneal crosslinking. Multiple biomechanical experiments have shown that corneal collagen crosslinking increases intra- and inter-fibrillar collagen bonds, thereby enhancing corneal stiffness as demonstrated by an increased Young’s elastic modulus (ratio of stress over strain) [18]. Our preliminary studies using ex vivo porcine tarsal plate demonstrated a gross difference in stiffness between control and treatment specimens, with treatment specimens having greater stiffness. However, these macroscopic changes were difficult to quantify, and gross photographs are clearly limited by numerous factors such as a lack of standardization of tissue alignment within the forceps and photography angle. We aimed to standardize the gross photographs using a metal clamp mounted in a fixed location relative to a tripod-mounted camera and a registration image (Fig. 2a). The measured change in “flop” was significantly different between control and treatment groups, with treatment specimens demonstrating smaller angles of variation from 180 degrees after treatment, presumably due to greater tissue stiffness (Fig. 2b).

We demonstrated validity of our tarsal crosslinking protocol using AS-OCT imaging, which to our knowledge has not been previously described for tarsal plate tissue. We observed a hyperreflective band at the anterior surface of crosslinked porcine specimens (Fig. 3). This band was present in 30-min treatment specimens, and its area was significantly greater (*P* = 0.003) and average intensity significantly brighter (*P* = 0.039) in 60-min treatment specimens, suggesting time-dependent correlation of the hyperreflective band with crosslinking treatment. A similar hyperreflective band was also observed in human tissue (Fig. 4). A hyperreflective demarcation line on AS-OCT imaging has been described in crosslinked corneal tissue as the proposed transition between the crosslinked anterior stroma and untreated posterior stroma. It has become widely accepted as a biomicroscopic marker to monitor the depth of effective corneal crosslinking treatment [26]. While this is the first time that AS-OCT imaging has been used to reveal structural details of tarsus, it is interesting that an analogous hyperreflective line was noted with our crosslinking treatment, which may indicate a similar demarcation between the crosslinked anterior tarsal plate and untreated posterior aspect. This OCT finding warrants further exploration, as this may be a noninvasive technique for tracking crosslinking safety (i.e. monitoring depth of penetrance) and efficacy (i.e. extent of crosslinking) in future in vivo tarsal crosslinking procedures, especially with the increased use of intraoperative OCT imaging.

We sought to further quantify the changes observed in porcine and human tarsal plate tissue by performing uniaxial tensile testing. Young’s modulus values indicate the elasticity of the tissue, while strain at break and peak stress measure tissue strength. Unfortunately, these studies were limited by small sample size and tissue slippage during testing, and no significant difference was noted in tensile properties in porcine and human samples. Further study with larger sample sizes is needed. Smith and colleagues have recently demonstrated a significant increase in tissue stiffness by tensile testing using a short-duration, high-irradiance crosslinking protocol in sheep tarsal plate [21]. Our experimental protocol utilized a lower irradiance UVA treatment, the safety of which has been established in some accelerated corneal crosslinking protocols [27]. It is unclear whether our results lacked statistical significance due to small sample size compounded with high biological variability, or whether higher UVA irradiance during crosslinking is necessary for maximal stiffening effect.

There are several drawbacks to this study. The human tarsal plate samples were obtained from patients with floppy eyelid syndrome, however, the diagnosis of this condition is difficult to standardize from provider to provider. It remains to be seen whether the observed tarsal plate stiffening would apply in non-FES tarsal plate tissue. Ideally, internal control samples would be taken from the same tissue specimen, however this was not possible due to the small tissue size and the specimen size constraints of the available tensile testing apparatus. Also, tarsal plate specimens in this study were crosslinked with conjunctiva dissected off. In vivo, an anterior surgical approach to the tarsal plate through an eyelid crease incision would allow crosslinking treatment with conjunctiva off. A posterior approach to the eyelid for crosslinking would allow a surgical incision to be avoided, but it remains unclear whether results would differ significantly with conjunctiva on. There are limitations to the observations made, as the clinical relevance of the flop measurements and the observed OCT hyperreflective band are not known. Further studies with greater sample sizes in both ex vivo animal tissue and in vivo human tissue are indicated to determine crosslinking parameters with optimal efficacy and preserved safety. In addition, a previous study demonstrated that there is a lack of structural change on histopathology of tarsal plate tissue after crosslinking treatment [21]. However, studies using electron microscopy would be beneficial for further analysis in the future.

## Conclusions

Altogether, our results imply efficacy of tarsal crosslinking in increasing stiffness in both porcine tarsus and human tarsus from FES patients. Tarsal collagen crosslinking in porcine and human tissue has not been previously described, so our study provides proof-of-principle for a novel treatment protocol for FES. We also describe a unique use of AS-OCT for tarsal plate imaging and potentially for monitoring tarsal crosslinking safety and efficacy. Our results demonstrated a clear difference in tissue stiffness with gross photographs (“flop”), but tensile testing did not demonstrate a significant difference in tensile properties. Further evaluation with tensile testing is necessary with larger sample sizes. Should future studies continue to demonstrate increased biomechanical stiffness as a result of tarsal collagen crosslinking, investigation of this procedure in humans as a minimally invasive treatment for FES may be warranted.

## Data Availability

The datasets used and/or analyzed during the current study are available from the corresponding author on reasonable request.

## Abbreviations

AS-OCT: Anterior segment optical computed tomography
BAK: Benzalkonium chloride
CPAP: Continuous positive airway pressure
FES: Floppy eyelid syndrome
MMP: Matrix metalloproteinases
OSA: Obstructive sleep apnea
SD: Standard deviation
UVA: Ultraviolet A
